# Unraveling the Association of Liver Steatosis and Fibrosis with Vitamin B12: A Cross-Sectional Study

**DOI:** 10.3390/metabo14110618

**Published:** 2024-11-12

**Authors:** Silvia Espina, Diego Casas-Deza, Vanesa Bernal-Monterde, Ana Royo-Esteban, Maria Pilar García-Sobreviela, Pilar Calmarza, Ana B. Martinez-Martinez, Jesús Osada, Jose M. Arbones-Mainar

**Affiliations:** 1 Gastroenterology Department, Miguel Servet University Hospital, 50009 Zaragoza, Spain; sespina@salud.aragon.es (S.E.); dcasas@salud.aragon.es (D.C.-D.); vbernal@salud.aragon.es (V.B.-M.); amroyoe@salud.aragon.es (A.R.-E.); 2 Adipocyte and Fat Biology Laboratory (AdipoFat), Translational Research Unit, Miguel Servet University Hospital, 50009 Zaragoza, Spain; mpgarcia@iisaragon.es (M.P.G.-S.); amarmar@unizar.es (A.B.M.-M.); 3 Instituto de Investigacion Sanitaria (IIS) Aragon, 50009 Zaragoza, Spain; josada@unizar.es; 4 Clinical Biochemistry Department, Miguel Servet University Hospital, 50009 Zaragoza, Spain; mpcalmarza@salud.aragon.es; 5 Centro de Investigacion Biomedica en Red Enfermedades Cardiovasculares (CIBERCV), Instituto Salud Carlos III, 28029 Madrid, Spain; 6 Facultad de Ciencias de la Salud, Universidad de Zaragoza, 50009 Zaragoza, Spain; 7 Departamento de Bioquímica y Biología Molecular y Celular, Facultad de Veterinaria, Instituto Agroalimentario de Aragón, CITA-Universidad de Zaragoza, 50013 Zaragoza, Spain; 8 CIBER Fisiopatología Obesidad y Nutricion (CIBERObn), Instituto Salud Carlos III, 28029 Madrid, Spain; 9 Instituto Aragones de Ciencias de la Salud (IACS), 50009 Zaragoza, Spain

**Keywords:** transient elastography, cirrhosis, NAFLD, fatty liver

## Abstract

Background: There are conflicting studies reporting both an increase and a decrease in vitamin B12 (VB12) levels in non-alcoholic fatty liver disease (NAFLD). In this study, we aimed to dissect the effects of steatosis and fibrosis on VB12. Methods: This is a cross-sectional study including all patients with a vibration-controlled transient elastography (VCTE) performed at the Hospital Miguel Servet (Zaragoza, Spain) between 2019 and 2022 for a chronic liver disease and having a recent blood test for VB12 levels. Liver fibrosis was assessed by VCTE and hepatic steatosis by ultrasonography and/or through controlled attenuation parameter (CAP). Results: 1195 patients (NAFLD *n* = 441, other chronic liver disease *n* = 754) were included. Median age was 57 years, 53% female. Patients with NAFLD had lower levels of VB12 compared to the rest of chronic liver diseases (289 vs. 313 pg/mL, *p* < 0.001). A significant negative correlation was observed between VB12 levels and hepatic steatosis measured by CAP (*r* = −0.13, *p* < 0.001). A significant positive correlation was observed between VB12 levels and liver stiffness in patients with NAFLD in both sexes (men *r* = 0.31, *p* < 0.001 and women *r* = 0.15, *p* = 0.016). A significant association between VB12 levels and liver fibrosis in cirrhosis stage was observed in patients with NAFLD (OR 1.06, 95% CI, 1.025–1.098, *p* = 0.001). Conclusion: VB12 levels were lower with greater hepatic steatosis. In NAFLD, VB12 levels were lower compared to other chronic liver diseases but their levels increased with higher liver stiffness and in cirrhosis stage.

## 1. Introduction

Hepatic steatosis is characterized by the accumulation of intrahepatic fat exceeding 5% of liver weight [1]. It is mainly caused by two predominant forms of fatty liver disease: alcohol-induced fatty liver disease (AFLD) and non-alcoholic fatty liver disease (NAFLD) [2]. AFLD disease arises from chronic and detrimental alcohol consumption [3], affecting approximately 2–2.5% of the general population [4]. NAFLD represents the hepatic manifestation of metabolic syndrome and has emerged as the most prevalent cause of chronic liver disease in developed countries [5,6]. It affects around 25% of the global population due to the obesity epidemic and sedentary lifestyles [5]. Both conditions encompass a spectrum of liver damage, ranging from isolated hepatic steatosis to liver cirrhosis, and are associated with increased risks of major cardiovascular events and overall mortality [7,8]. Notably, NAFLD-related mortality is primarily attributed to cardiovascular disease, followed by extra-hepatic cancer and liver disease [9,10]. Importantly, the severity of liver fibrosis exponentially amplifies liver-related mortality [10].

Cobalamin or vitamin B12 (VB12) plays a critical role in the development of red blood cells, neurons, brain functions, and DNA formation [11]. It is intricately involved in the metabolism of homocysteine (Hcy), and elevated Hcy levels are associated with increased cardiovascular risk due to vascular endothelial damage [12]. As an essential vitamin, VB12 is exclusively obtained through the diet and is found in poultry, beef, fish, dairy products, and oral supplements [13].

Although regular alcohol consumption is believed to impair VB12 absorption [14], studies present conflicting findings regarding VB12 levels in chronic alcohol users without liver damage [15,16,17,18,19]. In contrast, chronic alcoholic liver disease tends to elevate VB12 levels [19,20]. In NAFLD, VB12 levels are consistently lower compared to healthy individuals, with a negative correlation observed between VB12 levels and the grade of fatty liver [21]. Studies examining the association between VB12 levels and liver fibrosis in NAFLD have yielded conflicting outcomes. Some indicate lower VB12 levels with increasing liver fibrosis stage [22,23], while others report no significant changes or even increased VB12 levels with greater fibrosis severity [24,25]. These discrepancies may be attributed to variations in the assessment of liver fibrosis, including the use of liver biopsy or non-invasive biomarkers [22,23,24,25]. Alternatively, the potential influence of underlying steatosis on the relationship between VB12 levels and liver fibrosis has been largely overlooked, and this may account for some of the reported inconsistencies.

This study will examine the relationship between VB12 levels and liver fibrosis, assessed using vibration-controlled transient elastography (VCTE), a method largely unexplored in NAFLD [26]. We aim to untangle the effects of steatosis and fibrosis on VB12 levels and compare these findings with other chronic liver diseases. Our goal is to provide a comprehensive understanding of VB12′s role in NAFLD and its implications for therapeutic strategies and clinical management.

## 2. Materials and Methods

### 2.1. Study Design and Participants

This is a cross-sectional study performed at the University Hospital Miguel Servet (Zaragoza, Spain). The inclusion criteria included having a VCTE between January 2019 and December 2022 and a blood test with the measurement of VB12 levels with an interval of less than 12 months from VCTE. If the same patient had several VCTEs, the one closest to the VB12 determination was chosen.

This study was conducted in compliance with the Ethical Principles for Medical Research Involving Human Subjects outlined in the Helsinki Declaration in 1975 (revised in 2000) and was approved by the local ethics committee (CEIC-A, ref. PI22/558).

### 2.2. Outcome Measures

All patients underwent measurement of liver stiffness by VCTE (FibroScan^®^ 430 Mini, Echosens, Paris, France), a non-invasive technique to assess liver fibrosis. To be considered valid, at least 10 valid measurements were obtained for each patient and they must fulfill the following criteria: more than 60% of measurements were successful and the IQR/median was less than 30% [27]. Fibrosis cut-offs used were <7.5 kPa (F0-1) (no or mild fibrosis), 7.5–9.5 kPa (F2) (moderate fibrosis), 9.5–14 kPa (F3) (advanced fibrosis), >14 kPa (F4) (liver cirrhosis) [28].

The diagnosis of hepatic steatosis was made by ultrasonography and/or through the controlled attenuation parameter (CAP) value obtained by FibroScan. CAP cut-off values indicating steatosis were adapted from the study by Chan et al. as follows: < 263 dB/m (no steatosis) and ≥263 dB/m (steatosis) [29]. Patients were classified as having NAFLD if they met all of the following criteria: (1) clinical diagnosis of NAFLD or CAP ≥ 263 dB/m, (2) exclusion of other liver diseases, and (3) lack of significant alcohol consumption of >21 standard drink per week in men and >14 standard drinks per week in women [30]. Patients were classified as alcohol-related liver disease category if they fulfilled any of the following options: (a) clinical history of alcohol-related liver disease, or (b) a current significant alcohol consumption [31].

Data were collected retrospectively from electronic medical records and laboratory information systems. Demographic characteristics and clinical data included age, sex, alcohol intake, and metabolic factors such as high blood pressure (HBP), dyslipidemia (DLP), obesity (body mass index ≥ 30 Kg/m^2^) and diabetes mellitus type 2 (T2D). Hypertriglyceridemia and/or hypercholesterolemia were considered DLP. Biochemical determinations were performed at the Clinical Biochemistry Department in the Miguel Servet University Hospital, including VB12, folate, hemoglobin, as well as the red blood cells indices mean corpuscular volume (MCV) and mean corpuscular hemoglobin (MCH).

### 2.3. Statistical Analysis

A descriptive statistical analysis was conducted using percentages for categorical variables and medians with interquartile ranges [IQR] for continuous variables. Most variables did not meet the normality assumption, as determined by the Shapiro–Wilk test, so we used the Mann–Whitney test for quantitative comparisons. For consistency, the Mann–Whitney test was also applied to variables meeting normality. Chi-squared tests were used for qualitative variable comparisons. The correlation between quantitative variables was studied using the Pearson correlation coefficient (*r*). Odds ratios (OR) adjusted for sex and age were obtained with 95% confidence intervals (95% CI) and a *p*-value < 0.05 was considered to indicate significance. Statistical analysis was carried out in R statistical software (v.4.1.2) and the appropriate packages.

## 3. Results

### 3.1. Baseline Characteristics

The study enrolled 1195 patients who underwent VCTE between January 2019 and December 2022. All of these patients had a VB12 determination within 12 months before or after the elastography assessment. The median lag between VCTE and VB12 determination was 2.2 months. Table 1 lists all chronic liver diseases included in the study. All causes of chronic liver disease, except NAFLD, were grouped under the name “non-NAFLD group”. The most frequent cause of chronic liver disease was NAFLD (*n* = 441, 36.9%), followed by hepatitis C virus (HCV) (*n* = 222, 18.5%), hepatitis B virus (HBV) (*n* = 134, 11.2%), cholestatic liver disease, which includes primary biliary cholangitis and primary sclerosing cholangitis (*n*= 133, 11.1%), and alcohol-related liver disease (*n* = 113, 9.4%).

The median age was 57 years [range 15–88] and 53% were women (Table 2). A percentage of 39.2% of the individuals suffered from obesity and 38.7% from DLP with no differences between the sexes. Compared to women, men had a higher prevalence of HBP (41.4% vs. 33.3%, *p* = 0.005) and T2D (27.4% vs. 21.5%, *p* = 0.021).

Men had higher median liver stiffness and hepatic steatosis than women (7.3 vs. 6.1 Kpa and 274 vs. 257 dB/m respectively, both *p* < 0.001), leading to more male patients suffering from severe liver fibrosis (F4) compared to women (20.8% vs. 12.1%, *p* < 0.001). However, the incidence of NAFLD was higher in women than in men (40.0% vs. 33.5%, *p* = 0.02).

Lastly, we did not observe sex-differences in terms of circulating levels of VB12 with a median value of 303 pg/mL, however women had increased folic acid concentrations compared to men (7.95 vs. 7.00 ng/mL, *p* < 0.001). Vitamin B12 and folic acid play essential roles in hemoglobin synthesis. As expected, levels of circulating hemoglobin were higher in men compared to women (14.8 vs. 13.7 g/dL, *p* < 0.001). Likewise, the median values of the red blood cells indices MCV and MCH also appeared elevated in men compared to women (91.5 vs. 89.7 fL and 33.6 vs. 33.3 pg, both *p* < 0.001).

### 3.2. NAFLD vs. Non-NAFLD: Clinical and Analytical Comparison

Table 3 describes and compares the main clinical and analytical characteristics of the patients with NAFLD vs. their non-NAFLD counterparts. Liver stiffness (fibrosis) did not differ between the NAFLD and non-NAFLD group (6.5 kPa for both groups). As expected, CAP values were significantly higher in the NAFLD vs. non-NAFLD group (321 vs. 237 dB/m, *p* < 0.001), reflecting greater hepatic fat accumulation. Patients with NAFLD, though younger, had more frequent metabolic comorbidities, including obesity, hypertension, type 2 diabetes, and dyslipidemia.

The study revealed a significant decrease in VB12 levels in the NAFLD group compared to the non-NAFLD group (289 vs. 313 pg/mL, *p* < 0.001). The NAFLD group also showed a lower mean corpuscular volume (MCV) compared to non-NAFLD patients (89.4 vs. 91.2 fL, *p* < 0.001), though mean corpuscular hemoglobin (MCH) remained the same in both groups.

Folic acid, another critical factor in this process, did not differ between the groups, suggesting that the MCV reduction is more reflective of NAFLD-associated metabolic changes than a direct effect of reduced B12. These findings, consistent across both male and female patients, underscore the impact of NAFLD on VB12 levels and related hematological parameters.

### 3.3. Correlation Between VB12 Levels, Hepatic Steatosis, and Liver Fibrosis in NAFLD and Non-NAFLD Patients

Table 4 compares clinical characteristics between patients with non-NAFLD and NAFLD, subdivided by fibrosis stages (F0-F1, F2-F3, and F4). NAFLD patients with advanced fibrosis (F4) were older than those in the early stages (F0-F1). As expected, liver stiffness and CAP values increased significantly with advancing fibrosis in NAFLD, indicating greater liver damage and fat accumulation. No significant differences in folic acid levels were observed between groups. VB12 levels in early-stage NAFLD (F0-F1) patients were significantly lower than in their non-NAFLD counterparts. In contrast, although the difference in B12 levels between early fibrosis (F0-F1) and advanced fibrosis (F4) did not reach statistical significance (*p* = 0.070), there is a clear trend of increasing B12 levels as fibrosis advances, with the highest levels seen in cirrhotic patients (F4).

**Figure 1:**
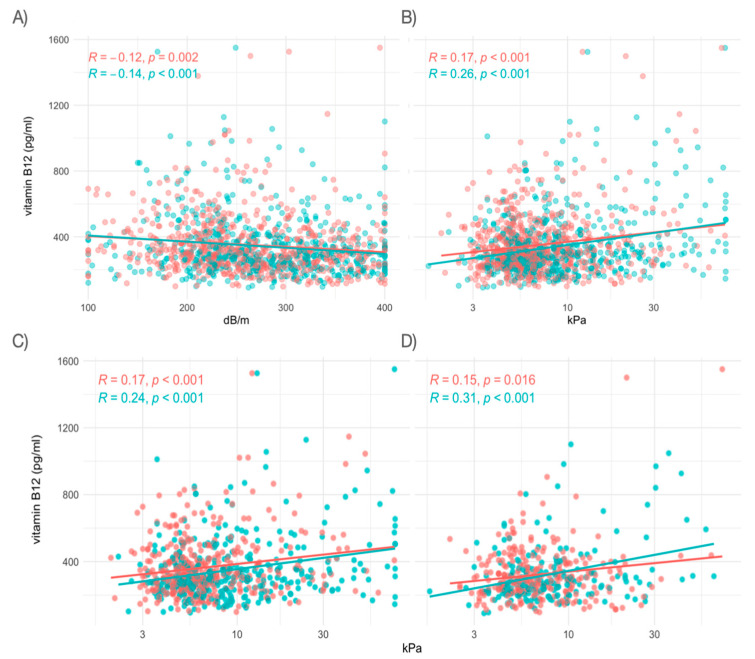
Correlation analysis. (**A**) Scatterplot of VB12 levels and hepatic steatosis measured as the controlled attenuation parameter (CAP) value obtained by vibration-controlled transient elastography (VCTE). (**B**–**D**) Scatterplot of VB12 levels and liver stiffness measured by VCTE in all patients (**B**), non-NAFLD group (**C**), and NAFLD group (**D**). Each point represents one individual (women in red and men in green). R: Pearson correlation coefficient.

To test these changes in a global manner, we performed a correlation analysis (Figure 1). First, we analyzed the correlation between VB12 levels and liver steatosis, as measured by CAP, and found a significant negative correlation in the entire cohort (*r* = −0.13, *p* < 0.001). This suggests that higher hepatic steatosis is associated with lower VB12 levels. This association was consistent across sexes, with correlations of *r* = −0.14 (*p* < 0.001) in men and *r* = −0.12 (*p* = 0.002) in women (Figure 1A).

Next, we examined the relationship between VB12 levels and liver fibrosis, observing a significant positive correlation in the entire cohort (*r* = 0.24, *p* < 0.001), with *r* = 0.26 (*p* < 0.001) in men and *r* = 0.17 (*p* < 0.001) in women (Figure 1B), indicating that VB12 levels increase with fibrosis severity.

Finally, we assessed the impact of steatosis and liver stiffness on VB12 in both the NAFLD and non-NAFLD groups. In both groups, liver stiffness positively correlated with VB12 levels. In the non-NAFLD group, correlations were *r* = 0.24 (*p* < 0.001) for men and *r* = 0.17 (*p* < 0.001) for women (Figure 1C), while in the NAFLD group, the correlations were stronger, with *r* = 0.31 (*p* < 0.001) for men and *r* = 0.15 (*p* = 0.016) for women (Figure 1D). These findings indicate a positive relationship between liver stiffness and VB12 levels in both groups.

### 3.4. Association Between VB12 Levels and Severe Fibrosis: NAFLD Group vs. Other Liver Diseases

**Figure 2:**
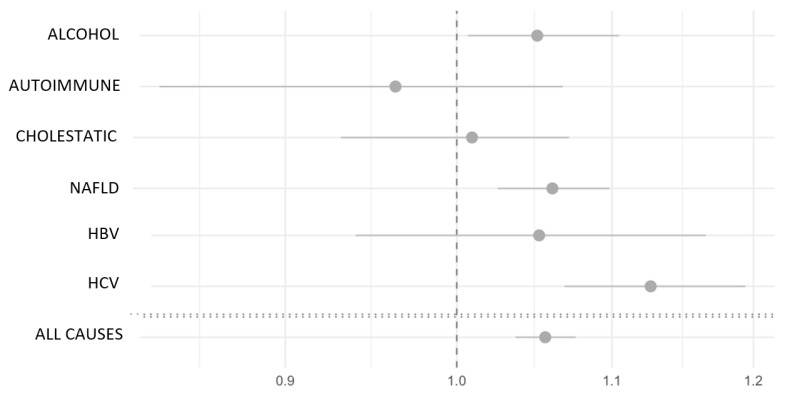
Odds ratios and 95% confidence intervals for the association between VB12 and liver cirrhosis (F4). ORs should be interpreted as the odds of having liver cirrhosis (F4) for a 25 pg/mL change in vitamin B12 adjusted for age and sex. NAFLD: non-alcoholic fatty liver disease, HCV: hepatitis C virus, HBV: hepatitis B virus.

We calculated odds ratios (ORs) to evaluate the association between VB12 levels and advanced liver cirrhosis (F4 stage) across various chronic liver diseases and in the overall cohort (Figure 2). Significant ORs were found in several groups. In the NAFLD group, the OR was 1.06 (95% CI, 1.03–1.10, *p* = 0.001), indicating that for every 25 pg/mL increase in VB12 levels, the odds of having cirrhosis (F4) increase by 6%. Similarly, in the HCV group, the OR was 1.13 (95% CI, 1.07–1.19, *p* < 0.001), reflecting a 13% increase in odds per 25 pg/mL increase in VB12. Alcohol-related liver disease also showed a significant association, with an OR of 1.05 (95% CI, 1.01–1.11, *p* = 0.035), indicating a 5% increase in the odds of F4 per 25 pg/mL rise in VB12.

In contrast, some groups exhibited non-significant ORs, though the trend towards increased risk of cirrhosis with higher VB12 levels persisted in cholestatic liver disease and HBV. Only autoimmune liver diseases deviated from this trend. When analyzing the overall cohort, combining all causes, the OR was 1.06 (95% CI, 1.04–1.08, *p* < 0.001), showing that higher VB12 levels consistently correlate with an increased likelihood of cirrhosis across all chronic liver diseases studied. These findings suggest a strong link between elevated VB12 levels and an increased risk of cirrhosis, particularly in NAFLD, HCV, alcohol-related liver disease, and the broader population of chronic liver disease patients.

## 4. Discussion

Vitamin B12 is stored primarily in the liver and its levels vary according to the type of chronic liver disease and the stage of liver fibrosis, with conflicting reports in NAFLD. Our study observes a distinct effect of steatosis and fibrosis on VB12 levels; liver fat accumulation correlates with lower VB12, while fibrosis is associated with higher VB12, independent of liver fat presence.

All subjects in our study had normal or borderline VB12 levels (>200 pg/mL) and normal folic acid levels (>4 ng/mL). Men had lower folate levels and non-significantly lower VB12 levels compared to women, which may explain their higher MCV and MCH values. VB12 and folate deficiencies are known causes of macrocytosis, defined by MCV values above 95–100 fL, typically accompanied by elevated MCH [32].

Previous studies reported lower VB12 levels in obese individuals and those with insulin resistance [33,34]. In addition, studies in patients with NAFLD showed that low VB12 levels are inversely correlated with triglycerides and cholesterol levels [35]. Consistent with this, our NAFLD group showed a high prevalence of metabolic factors: 58% obesity, 42.6% hypertension, 33.8% type 2 diabetes, and 48.3% dyslipidemia. As expected, VB12 levels were lower in the NAFLD group compared to non-NAFLD patients, despite similar levels of liver stiffness between the groups. These results were not influenced by the stage of fibrosis since both groups had similar liver stiffness. However, this decrease in VB12 was not accompanied by higher MCV or MCH values, likely due to the influence of alcohol-related liver disease in the non-NAFLD group, which is associated with elevated MCV [36]. The elevated plasma VB12 levels observed in patients with ALD, particularly cirrhosis, are likely due to decreased hepatic clearance and increased release from damaged liver cells. Lambert et al. reported that ALD patients show significantly higher levels of total plasma VB12, haptocorrin (HC)-bound VB12, and corrinoid analogs compared to controls; they also noted a positive correlation between cholestasis markers, such as serum alkaline phosphatase, and elevated levels of HC-bound VB12 and corrinoids in cirrhotic patients, suggesting that cholestasis contributes to the altered VB12 dynamics observed in ALD [37]. This elevation, notably in HC-bound VB12, is thought to result from impaired hepatic clearance, a consequence of liver dysfunction in advanced ALD stages [38]. Furthermore, the increased levels of homocysteine and methylmalonic acid, both indicators of functional VB12 deficiency, indicate that despite high plasma VB12 levels, cellular VB12 utilization may be impaired in ALD due to deficient cellular uptake or enzyme activation [39]. Kanazawa and Herbert also observed that alcoholics exhibit high plasma VB12 concentrations alongside low hepatic VB12 stores compared to non-alcoholics, suggesting that VB12 is retained in peripheral tissues but accumulates in plasma due to impaired hepatic processing [40]. This literature emphasizes a distinction between NAFLD and ALD: in ALD, liver dysfunction and clearance deficits appear central to VB12 alterations, whereas in NAFLD, associations with VB12 are more closely tied to metabolic syndrome factors and hepatic fat accumulation.

All NAFLD patients in our study had CAP values > 263 dB/m estimated by VCTE, confirming the presence of hepatic steatosis. Our analysis revealed a significant negative correlation between VB12 levels and CAP values in both men and women, indicating that higher hepatic steatosis is associated with lower VB12 levels. This finding aligns with previous research by Koplay M et al. [21] which also reported a negative correlation between VB12 levels and the severity of fatty liver. Our study is the first to demonstrate both a negative correlation between plasma vitamin B12 levels and CAP values, estimating liver fat accumulation, and a positive correlation between liver fibrosis assessed by VCTE and vitamin B12 levels in NAFLD patients. While some prior studies have reported associations between B12 levels and liver conditions, our use of VCTE provides a novel, non-invasive perspective, clarifying the differential impact of steatosis and fibrosis on B12 dynamics. These findings contribute to the broader understanding of micronutrient status in NAFLD and suggest that vitamin B12 could serve as a supplementary marker in liver health assessment, though further longitudinal studies are needed.

The relationship between serum VB12 levels and liver fibrosis is clear in some chronic liver diseases such as HCV or alcohol-related liver disease. In both, the levels of VB12 increase as liver fibrosis progresses [19,20,41,42]. In NAFLD, there are conflicting results regarding the relationship. Some studies show lower levels of VB12 as the liver fibrosis stage increased [22,23], other notice no changes [24], and others observe an increase in VB12 levels with the severity of liver fibrosis [25]. This variability may be due to the fact that the evaluation of liver fibrosis is performed either with a liver biopsy [22,24,25] or with non-invasive blood-based biomarkers [23]. Recently, a study has analyzed the levels of VB12 in individuals with NAFLD using VCTE to assess liver fibrosis, but a linear relationship between VB12 levels and the stage of liver fibrosis was not observed [43]. Our correlation analysis between VB12 levels and liver fibrosis measured by VCTE revealed a significant positive correlation in NAFLD and non-NAFLD patients, both in men and women. Thus, our findings indicate that liver fibrosis may have a stronger influence on circulating VB12 levels than hepatic steatosis. While both liver conditions impact VB12 dynamics, the correlation between VB12 levels and fibrosis (*r* = 0.24) was more pronounced than the correlation with steatosis (*r* = −0.13). Notably, the association between fibrosis and VB12 was strongest in men diagnosed with NAFLD (*r* = 0.31), suggesting that the progression of fibrosis might exacerbate changes in VB12 levels more than fat accumulation alone. These observations underscore the importance of considering fibrosis severity when evaluating VB12 levels in NAFLD and suggest that VB12 could potentially serve as a supplementary marker for fibrosis progression in clinical settings.

A study on viral liver diseases found that higher VB12 levels were significantly associated with worse survival outcomes in patients with chronic viral liver diseases, particularly in those with advanced fibrosis or cirrhosis [44]. Building on this, we expanded our investigation beyond viral liver diseases and NAFLD to assess whether this association applied more broadly across chronic liver diseases. We found a significant link between higher VB12 levels and cirrhosis (stage F4) in NAFLD, HCV, and alcohol-related liver disease, with a similar trend—though non-significant—in cholestatic liver disease and HBV. Only autoimmune liver diseases deviated from this pattern. These findings suggest that elevated VB12 levels are broadly associated with a higher risk of liver cirrhosis, underscoring the potential of VB12 as a marker of liver health across various chronic liver conditions.

Our study has several strengths. First, the large sample size and the use of VCTE to assess liver fibrosis, a highly accurate technique, add robustness to our findings. Additionally, CAP was employed to objectively estimate hepatic steatosis, further enhancing the study’s reliability. However, there are some limitations. The lack of participant follow-up prevents us from observing long-term changes. The observational design also limits our ability to establish a causal relationship between fibrosis progression and the increase in VB12 levels, and the monocentric nature of the study may introduce selection bias. Finally, the study lacks data on dietary intake, dietary management, prescribed medications, and BMI. These factors could influence vitamin B12 metabolism and hepatic function, potentially impacting the associations observed. Future studies incorporating these variables may provide a more comprehensive understanding of B12 dynamics in liver disease.

## 5. Conclusions

In summary, lower levels of VB12 were observed with greater hepatic steatosis and in NAFLD patients. VB12 levels were positively correlated with liver stiffness in NAFLD and the other chronic liver diseases, and higher VB12 levels were linked to an increased likelihood of liver cirrhosis in NAFLD, HCV, and alcohol-related liver disease. Although these findings indicate an association between B12 levels and liver fibrosis in NAFLD, they do not justify routine serial B12 monitoring as part of standard follow-up. Prospective studies are needed to determine whether B12 measurements could serve as a reliable biomarker for disease progression or treatment response in NAFLD and other liver conditions.

## Figures and Tables

**Table 1:** Liver diseases included in the study.

NAFLD group (*n* = 441)
Non-NAFLD Group (*n* = 754)
HCV (*n* = 222)
HBV (*n* = 134)
Cholestatic liver disease (*n* = 133)
Alcohol-related liver disease (*n* = 113)
Autoimmune hepatitis (*n* = 53)
Hemochromatosis (*n* = 19)
Wilson disease (*n* = 6)
Cryptogenic (*n* = 74)

NAFLD: non-alcoholic fatty liver disease, HCV: hepatitis C virus, HBV: hepatitis B virus.

**Table 2:** Baseline characteristics of the patients according to sex.

	Females (*n* = 637)	Males (*n* = 558)	All (*n* = 1195)	*p*-Value
Age (years)	58.0 [48.0; 68.0]	57.0 [48.0; 65.0]	57.0 [48.0; 66.0]	0.194
Obesity	245 (38.5%)	223 (40%)	468 (39.2%)	0.637
Dyslipidemia	248 (38.9%)	214 (38.4%)	462 (38.7%)	0.884
High blood pressure	212 (33.3%)	231 (41.4%)	443 (37.1%)	0.005
Diabetes mellitus	137 (21.5%)	153 (27.4%)	290 (24.3%)	0.021
NAFLD	255 (40.0%)	186 (33.3%)	441 (36.9%)	0.020
Liver fibrosis (kPa)	6.1 [4.7; 9.5]	7.3 [5.23; 11.8]	6.5 [4.9; 10.3]	<0.001
F4 stage	77 (12.1%)	116 (20.8%)	193 (16.2%)	<0.001
CAP (dB/m)	257 [214; 316]	274 [227; 330]	265 [221; 323]	<0.001
Vitamin B12 (pg/mL)	314 [236; 418]	290 [227; 393]	303 [232; 406]	0.059
Folic acid (ng/mL)	7.95 [5.66; 11.2]	7.00 [5.3; 10.1]	7.5 [5.5; 10.7]	<0.001
Hemoglobin (g/dL)	13.7 [12.8; 14.4]	14.8 [13.9; 15.7]	14.2 [13.2; 15.1]	<0.001
MCV (fL)	89.7 [86.2; 93.5]	91.5 [88.1; 95.0]	90.5 [86.8; 94.3]	<0.001
MCH (pg)	33.3 [31.9; 34.0]	33.6 [32.7; 34.3]	33.5 [32.2; 34.2]	<0.001

NAFLD: non-alcoholic fatty liver disease, F4: liver cirrhosis, CAP: controlled attenuation parameter, MCV: mean corpuscular volume, MCH: mean corpuscular hemoglobin.

**Table 3:** Baseline characteristics of the patients according to NAFLD presence.

	NAFLD Group (*n* = 441)	Non-NAFLD Group (*n* = 754)	*p*-Value
Age (years)	56.0 [47.0; 64.0]	58.0 [49.0; 67.0]	0.002
Obesity	256 (58.0%)	212 (28.1%)	<0.001
Dyslipidemia	213 (48.3%)	249 (33.0%)	<0.001
High blood pressure	188 (42.6%)	255 (33.8%)	0.003
Diabetes mellitus	149 (33.8%)	141 (18.7%)	<0.001
CAP (dB/m)	321 [273; 361]	237 [204; 284]	<0.001
Liver fibrosis (kPa)	6.50 [4.90; 9.80]	6.50 [4.90; 10.9]	0.505
Vitamin B12 (pg/mL)	289 [220; 384]	313 [240; 420]	<0.001
Folic acid (ng/mL)	7.61 [5.55; 10.8]	7.42 [5.39; 10.6]	0.711
MCV (fL)	89.4 [86.0; 93.0]	91.2 [87.7; 94.9]	<0.001
MCH (pg)	33.5 [32.2; 34.2]	33.5 [32.3; 34.2]	0.546

NAFLD: non-alcoholic fatty liver disease, CAP: controlled attenuation parameter, MCV: mean corpuscular volume, MCH: mean corpuscular hemoglobin.

**Table 4:** Comparison of clinical characteristics and Vitamin B12 levels across fibrosis stages in NAFLD and non-NAFLD patients.

	NAFLD			Non-NAFLD (*n* = 754)	*p*-Value (Mann–Whitney)	
	F0–F1 (*n* = 259)	F2–F3 (*n* = 124)	F4 (*n* = 58)		F0–F1 vs. F4	Non-NAFLD vs. F0–F1
Age (years)	54.0 [45.0; 62.0]	57.0 [48.0; 65.0]	62.0 [53.0; 69.0]	58.0 [49.0; 67.0]	<0.001	<0.001
Liver fibrosis (kPa)	5.20 [4.40; 6.05]	9.65 [8.60; 10.6]	20.8 [17.2; 28.8]	6.50 [4.90; 10.9]	<0.001	<0.001
CAP (dB/m)	312 [270; 355]	328 [276; 363]	332 [297; 379]	237 [204; 284]	0.020	<0.001
Folic acid (ng/mL)	7.60 [5.67; 10.9]	7.21 [5.45; 10.5]	8.45 [5.50; 10.6]	7.42 [5.39; 10.6]	0.650	0.565
Vitamin B12 (pg/mL)	273 [218; 370]	295 [220; 389]	312 [238; 437]	313 [240; 420]	0.070	<0.001

NAFLD: non-alcoholic fatty liver disease, CAP: controlled attenuation parameter.

## Data Availability

The data presented in this study are available on request from the corresponding author upon Ethical Committee approval (CEICA, https://www.iacs.es/investigacion/comite-de-etica-de-la-investigacion-de-aragon-ceica/ceica-evaluaciones-y-otras-presentaciones/, accessed on 7 November 2024).
